# Carpal Tunnel Release with Ultrasound Guidance Versus Open and Mini-Open Carpal Tunnel Release: A Systematic Review and Meta-Analysis of Randomized Controlled Trials

**DOI:** 10.1016/j.jhsg.2024.04.001

**Published:** 2025-01-20

**Authors:** Seper Ekhtiari, Mark Phillips, Dalraj Dhillon, Ali Shahabinezhad, Conner McMains, Bill Dzwierzynski, Mohit Bhandari

**Affiliations:** ∗ Orthopaedic Surgery, Addenbrooke’s Hospital, Cambridge University Hospitals NHS Foundation Trust, Cambridge, Cambridgeshire, UK; † Department of Surgery, McMaster University, Hamilton, Canada; ‡ Faculty of Health Sciences, McMaster University, Hamilton, Canada; § Department of Family Medicine, University of Ottawa, Ottawa, Canada; ‖ Department of Plastic Surgery, Medical College of Wisconsin, Milwaukee, WI

**Keywords:** Carpal tunnel release, Meta-analysis, Ultrasound-guided

## Abstract

**Purpose:**

Carpal tunnel release (CTR) can be performed using several techniques, including traditional open CTR, mini-open CTR, endoscopic CTR (ECTR), and CTR with ultrasound guidance (CTR-US). Carpal tunnel release with ultrasound guidance allows the procedure to be performed through a small, nonpalmar incision while maintaining visualization of critical anatomy and may confer benefits in terms of early recovery and incision-related complications. The objective of this study was to compare CTR-US with traditional open or mini-open CTR based on evidence from randomized controlled trials (RCTs).

**Methods:**

The electronic databases Embase and MEDLINE were searched from inception to November 2022. Randomized controlled trials comparing CTR-US with traditional open or mini-open CTR were eligible for inclusion. Studies were assessed for eligibility from title and abstract followed by a full-text review. The main outcomes of interest were return to normal activity or return to work, patient-reported functional scores, and complications.

**Results:**

Three RCTs were eligible for inclusion with a total of 221 patients randomized. Meta-analysis demonstrated that compared with open CTR patients, patients treated with CTR-US had significantly higher functional scores at 3 months (standardized mean difference: −0.91, 95% confidence interval (CI): −1.38 to −0.44, *P* < .01) and faster return to normal activities (mean difference: −20.8 days, 95% CI: −21.77 to −19.73). There was no significant difference in complication rates between the two groups (odds ratio: 0.80, 95% CI: 0.04–15.10, *P* = .07). No domains were deemed to be at high risk of bias in any study.

**Conclusions:**

Based on the available evidence, CTR-US is a safe and effective surgical option for treating carpal tunnel syndrome with a similar risk profile to open CTR. Data suggest that patients who receive CTR-US have improved functional outcomes and faster return to work or normal activities. Future RCTs with larger sample sizes are needed to corroborate these benefits and demonstrate long-term outcomes of CTR-US.

**Type of study/level of evidence:**

Therapeutic II.

Carpal tunnel syndrome (CTS) is the most common compressive neuropathy, with an approximately 3% prevalence in the United States.^1^ The condition has a female-to-male predominance of approximately 3–1 and most often occurs in the fourth to sixth decades of life.^2^ Diabetes mellitus, obesity, pregnancy, and hypothyroidism are medical risk factors for developing CTS. Working with high force, high repetition, or vibrating tools is an occupational risk for developing CTS.^3^ Nonsurgical treatment modalities include splinting and nonsteroidal anti-inflammatory medications.^4^ Splinting is low-cost and well-tolerated; patients using nighttime splints are more than three times more likely to experience improvement in symptoms than those with no treatment. No splint design has been shown to be superior. Corticosteroid injections are often used for symptomatic management and may delay the need for surgery but do not treat the underlying disease process.^4^^,^^5^ Although many patients report resolution of symptoms when managed conservatively, some do not respond completely. Patients who fail conservative treatment may be indicated for carpal tunnel release (CTR), a procedure that is the definitive treatment for CTS.^5^

The most important element of CTR is increasing the space in the carpal tunnel through complete release of the transverse carpal ligament, which can be achieved by several techniques.^6^, ^7^, ^8^ Traditional open CTR is generally performed through a 3–8 cm palmar incision that may cross the distal wrist crease, whereas mini-open CTR utilizes a slightly smaller incision (open [OCTR] and mini-open CTR referred to as mOCTR), which carries the risks of pillar pain, injury to the palmar cutaneous branch of the median nerve, delayed wound healing, and hypersensitive scar. These complications have been associated with prolonged recovery periods that delay return to work or normal activity.^5^^,^^6^

The mini-open technique was developed following the adoption of endoscopic CTR (ECTR). In this technique, the transverse carpal ligament is released through a smaller 1–3 cm palmar incision that does not cross the distal wrist crease. Although this reduces the risk of damage to the palmar cutaneous branch and limits scar-related complications at a flexion crease, the incision is still located in the palm, which carries a continued risk of pillar pain and prolonged scar tenderness or hypersensitivity. Furthermore, the smaller incision may limit the surgeon’s visualization of critical anatomy during the procedure.

Like ECTR, carpal tunnel release with ultrasound guidance (CTR-US) can also be performed through a single, small, subcentimeter, nonpalmar incision. The high image resolution with contemporary ultrasound confers the ability for the surgeon to locate delicate structures in real time. Ultrasound with a small incision allows complete release of the transverse carpal ligament while maintaining a clear view of critical structures.^9^^,^^10^

A previous systematic review of 20 studies on CTR-US concluded that CTR-US may be an effective treatment for CTS with a short recovery period.^9^ However, these studies were based on a low level of evidence; 14 of 20 studies cited were case series, and there was no quantitative synthesis of the data. Thus, the purpose of this systematic review and meta-analysis was to compile the available data from randomized controlled trials (RCTs) comparing CTR-US with open CTR approaches. Meta-analyses were performed on patient function, time to return to normal activities, and complications.

## Methods

This study was conducted according to the Cochrane Handbook of Systematic Reviews of Interventions Version, Sixth Edition.^11^ The study is reported according to the Preferred Reporting Items for Systematic Reviews and Meta-Analyses statement.^12^

### Eligibility criteria

Randomized controlled trials comparing CTR-US with OCTR and/or ECTR were included. Studies were limited to English language publications. Systematic or narrative reviews, lower level of evidence clinical studies, commentaries, or editorials were excluded.

### Information sources and search strategy

The electronic databases MEDLINE and Embase were searched from the oldest available date to November 2022. The search strategy used included the key terms “carpal tunnel syndrome” and “carpal tunnel release.” The full search strategy can be found in Appendix 1 (available online on the *Journal’s* website at https://www.jhsgo.org).

### Selection process

Study titles and abstracts were screened for eligibility for inclusion. Any potentially eligible study underwent a full-text review, at which point, it was determined whether to include or exclude the study. Any questions about eligibility were discussed among the authors. The study selection was performed using Covidence (Veritas Health Innovation).

### Data collection process and data items

Data were collected using a spreadsheet from the full texts of all included articles. For data used in meta-analyses, the accuracy was verified at the analysis stage. Study characteristics were recorded, including study design, level of evidence, sample size, and country of origin. Descriptive patient data were also extracted, including mean patient age, distribution by sex, and number of patients allocated to each treatment group (if applicable). Any outcomes reported were also extracted including time point of collection, measure of central tendency, and measure of dispersion as applicable. Surgery type was defined as per the original author’s description.

### Study risk of bias assessment

Risk of bias for the included studies was assessed using the Cochrane Risk of Bias Assessment Tool.^13^

### Outcomes, effect measures, and synthesis methods

The main outcomes of interest were return to activity (as reported by the study, including “normal activity” or “return to work”), patient-reported functional scores, and complications. Continuous data were synthesized using mean difference if all studies used the same outcome instrument. If studies used different outcome instruments, standard mean difference was used. For dichotomous outcomes, odds ratios were calculated. All measures of effect are presented with their respective 95% confidence interval (CI). Random effects were used for all analyses to account for population and technique differences between studies, and *P* values of less than .05 were considered statistically significant.

### Reporting bias assessment

Given that, there were fewer than 10 studies included in all meta-analyses, and there were too few studies to perform a funnel plot analysis to assess for potential publication bias.^14^

## Results

### Study selection

**Figure 1:**
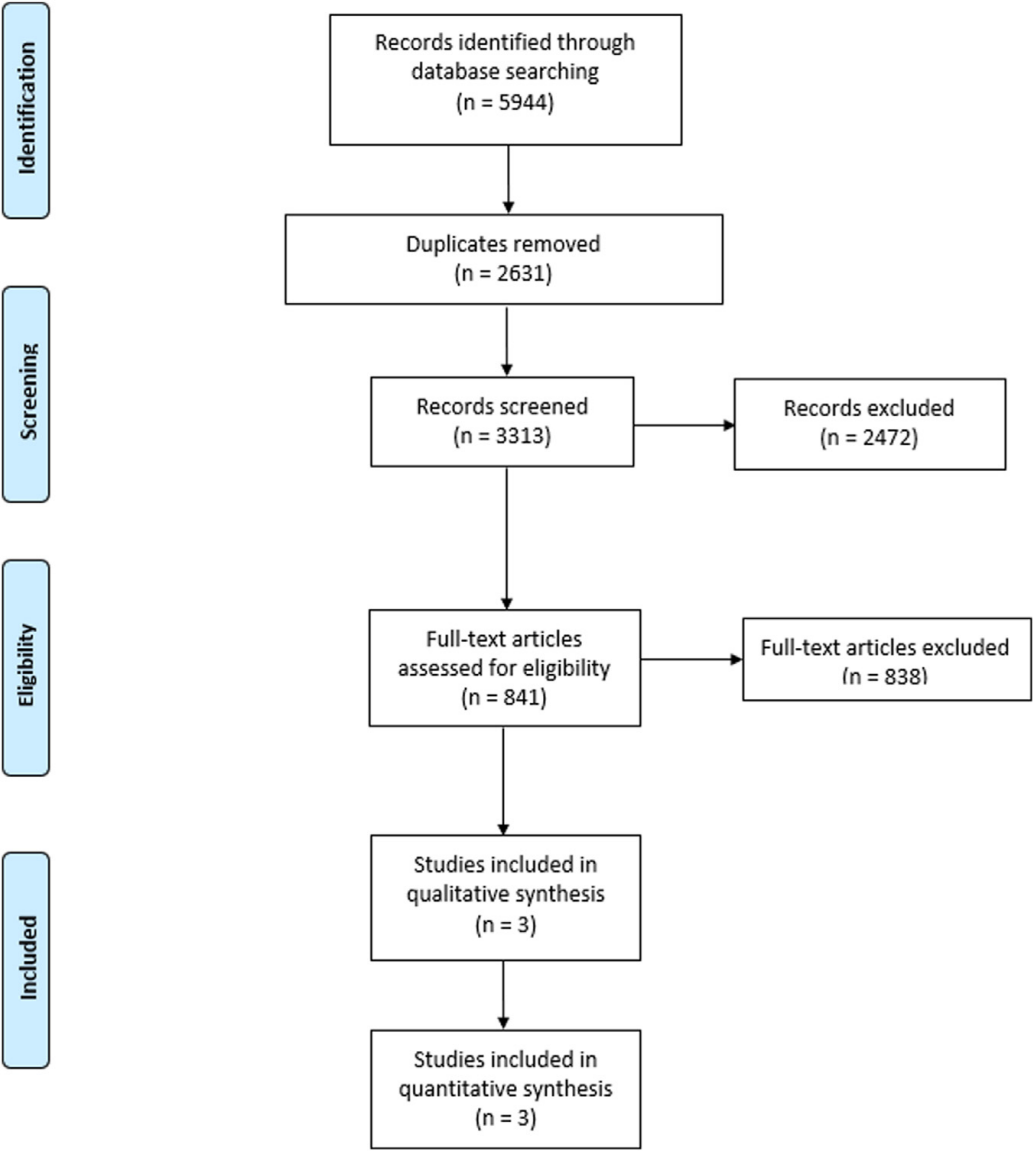
Study selection flow diagram.

A total of 5,944 references were identified by the literature search, with 2,631 duplicates being removed. Of these, 3,313 studies were screened against title and abstract, with 2,472 being excluded. Thus, 841 studies were assessed for full-text eligibility, and ultimately, three RCTs were included in this systematic review.^15^, ^16^, ^17^ Figure 1 contains the study selection flow diagram.

### Study characteristics

Two studies compared CTR-US with mini-open CTR, whereas the third study compared CTR-US with traditional OCTR. The three studies randomized a total of 221 patients—113 patients were randomized to CTR-US, 66 patients were randomized to mOCTR, and 42 to OCTR. All studies were performed in Spain and published in English. Table 1 summarizes the characteristics of the included studies.

**Table 1:** Characteristics of Included Studies

Primary Author	Year	Sample Size	Intervention	Comparator
Rojo-Manaute	2016	92	CTR-US	Mini-open CTR
Capa-Grasa	2014	40	CTR-US	Mini-open CTR
de la Fuente	2021	89	CTR-US	Open CTR

### Results of individual studies

One of the included articles was a pilot RCT study where the authors demonstrated the feasibility of an RCT comparing CTR-US with mini-OCTR.^17^ Rojo-Manaute et al subsequently performed a single operator, prospective, randomized trial with blinded outcome assessment between CTR-US and mOCTR.^15^ This trial collected outcomes at 3, 6, and 12 months, with no functional or pain outcomes reported at any time points earlier than 3 months. The authors reported that compared with mOCTR, patients receiving CTR-US had significantly better Quick Disabilities of the Arm, Shoulder, and Hand scores, for the first 6 months after surgery, as well as significantly faster return to normal daily activities or work (4.9 vs 25.4 days, *P* < .05). In addition, patients in the CTR-US group discontinued oral analgesics sooner and achieved complete wrist flexion and extension more quickly. From weeks 1 to 3 after surgery, patients in the CTR-US group reported lower pain. There were no complications in the CTR-US group, whereas there were two cases of complex regional pain syndrome and one superficial infection in the mOCTR group.

In their RCT, de la Fuente et al compared CTR-US and OCTR in a working population.^16^ Two separate surgeons performed the either CTR-US or OCTR procedures, respectively; both of whom had over 20 years of experience treating CTS. The authors reported significantly lower pain scores at 3 months of follow-up in the CTR-US group compared with mOCTR, as well as significantly better scores on the Boston Carpal Tunnel Syndrome Questionnaire Function Score, although this difference did not reach the minimal clinically important difference. No significant difference between the groups was found across a range of other outcomes, including days off work, Boston Carpal Tunnel Syndrome Questionnaire symptom scores, grip strength, time to becoming asymptomatic, nerve conduction studies, pinch grip strength, or complication rates.

### Syntheses of results

**Figure 2:**
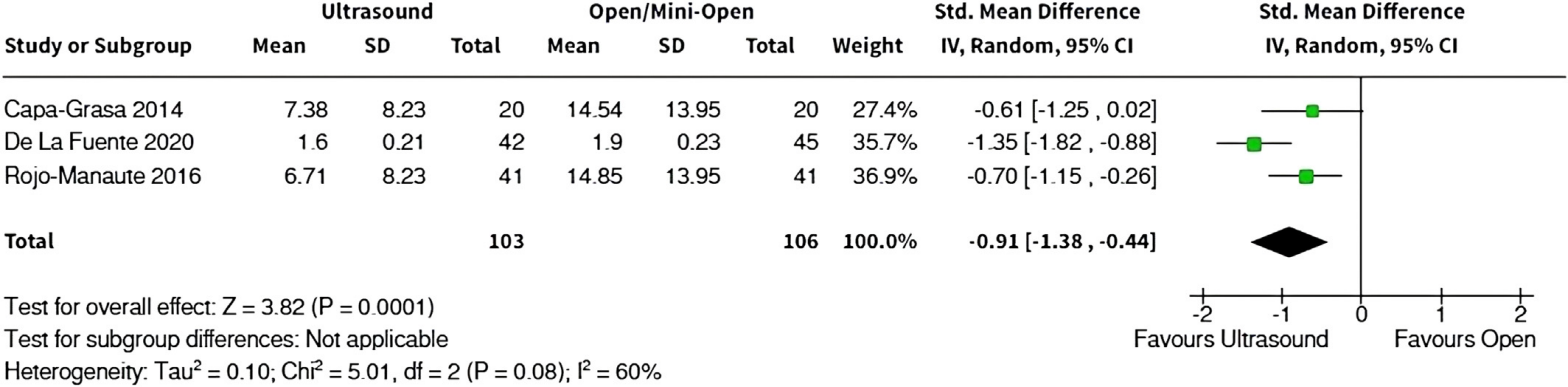
Forest plot of patient-reported function.

**Figure 3:**
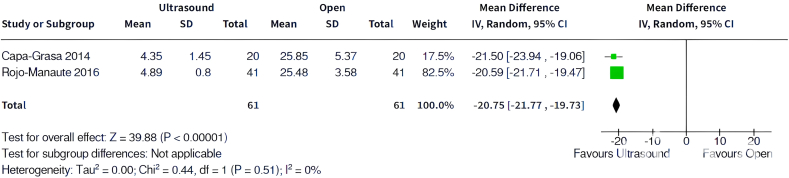
Forest plot of time to return to normal activities.

Three meta-analyses were performed on the outcomes of interest: function, return to normal activities, and complications. In terms of patient-reported function, the meta-analysis (Fig. 2) found that patients in CTR-US group had significantly better functional scores compared with OCTR patients (standard mean difference: −0.91, 95% CI: −1.38 to −0.44, *P* < .01). A meta-analysis of time to return to normal activities (Fig. 3) found that CTR-US patients returned to activities 3 weeks earlier than those who underwent OCTR (mean difference: −20.8 days, 95% CI: −21.77 to −19.73, *P* < .01).

**Figure 4:**
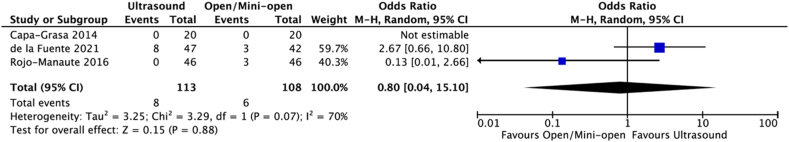
Forest plot of complications.

A total of 14 complications (6.3%) were reported across the three included studies—8 complications were reported in CTR-US patients (8/113, 7.1%) and 6 complications in the OCTR groups (6/108, 5.6%). A meta-analysis (Fig. 4) revealed no significant difference in overall complication rates between groups (odds ratio: 0.80, 95% CI: 0.04–15.10, *P* = .07). Complex regional pain syndrome occurred in three patients, all in the OCTR groups. There were two infections in OCTR groups, and one infection in the CTR-US groups; one superficial infection occurred in an mOCTR patient, whereas the depth/severity of the other two infections was not specified. Table 2 summarizes all reported complications by group.

**Table 2:** Reported Frequency of Complications by Group∗

Variable	CTR-US	Open/Mini-Open	Total
Persistent symptoms	2	0	2
Fibrotic scar proliferation	1	0	1
Cutaneous scar pathology	2	1	3
Hematoma	2	0	2
infection	1	2	3
CRPS	0	3	3
**Total**	8	6	14

∗ Definitions of complications as per the included articles.

### Risk of bias in studies

**Figure 5:**
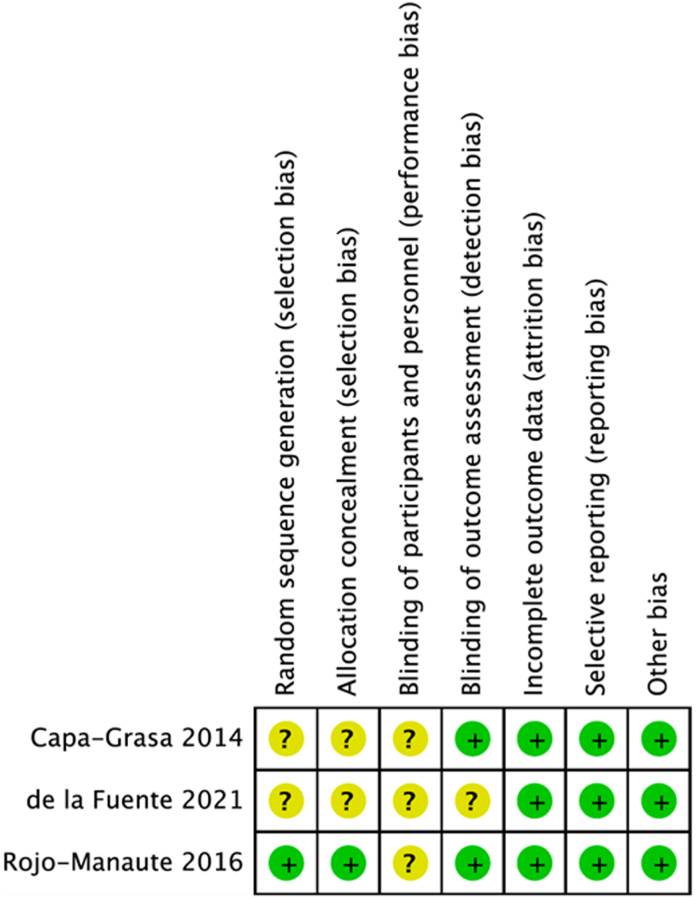
Risk of bias in individual studies.

**Figure 6:**
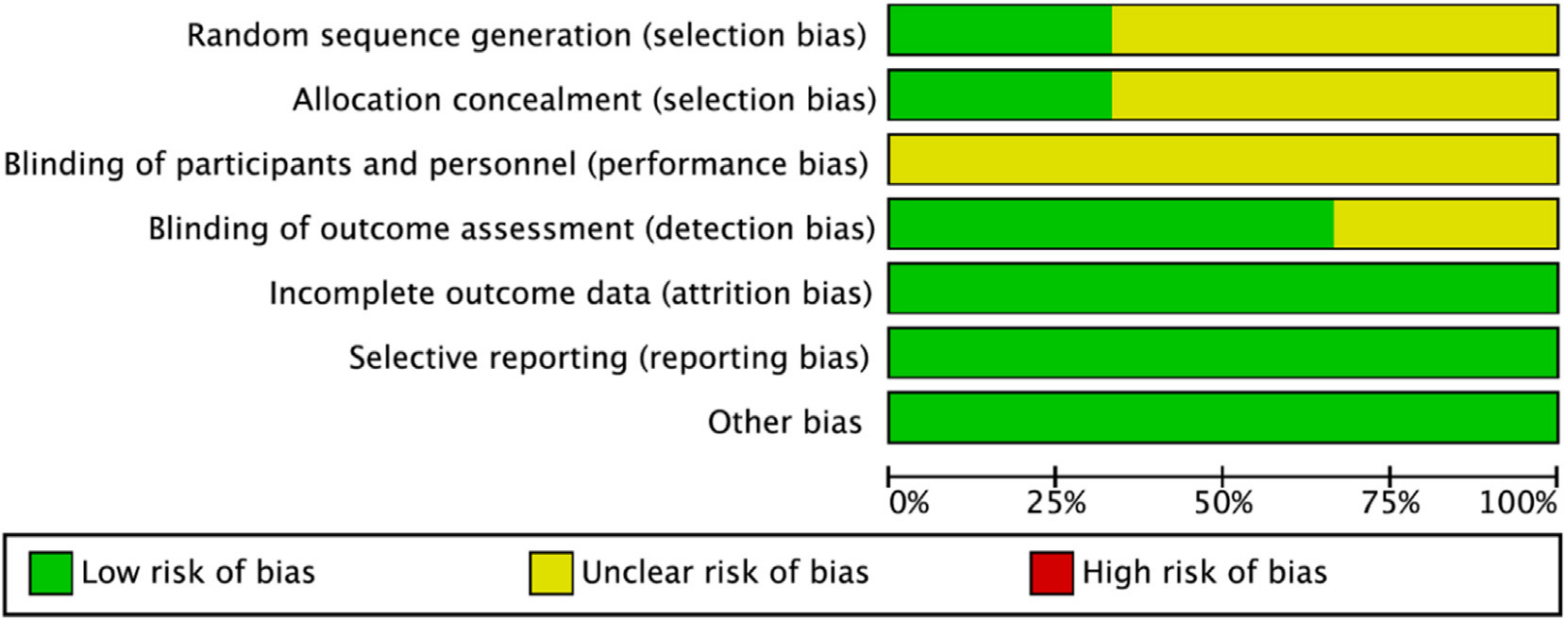
Risk of bias summary.

No domains were deemed to be at high risk of bias in any study. Attrition, reporting, and other biases were at low risk across all studies. Figures 5 and 6 summarize the risk of bias assessment across the included studies.

## Discussion

The results of this systematic review and meta-analysis of RCTs suggest that based on the best available evidence, patients undergoing CTR-US have superior early function, faster return to normal activities (including work), and no difference in complication rates when compared with the surgical gold standard of open CTR. We suggest that the improved results from CTR-US are in part due to release of the transverse carpal ligament through a nonpalmar incision without disruption of the skin, superficial fascia, and muscles in the palm.^8^

Complications rates were not different between CTR-US and OCTR in this analysis. The much shorter incision in CTR-US likely reduces the risk of postoperative pillar pain and scar tenderness.^1^, ^2^, ^3^, ^4^, ^5^, ^6^, ^7^, ^8^, ^9^, ^10^, ^11^, ^12^, ^13^, ^14^, ^15^, ^16^, ^17^ Within included studies, incision sizes for OCTR were 2–3 cm and 1–3 mm for CTR-US.^15^, ^16^, ^17^ Two patients in the CTR-US group had persistent symptoms that eventually required revision OCTR. Findings in these reoperative cases were not reported in the manuscript. The general complication rate for mini-open CTR, by comparison, is 2.6% at 18 months, and most often is due to superficial infection.^13^

Standard open CTR employs a relatively long incision spanning the proximal palm and distal wrist, whereas the mini-open technique utilizes a shorter incision at the cost of a narrower field of view. Although these procedures are accepted as safe, iatrogenic injury to the median nerve still rarely occurs. Carpal tunnel release with ultrasound guidance employs real-time ultrasound imaging guidance for the release of the transverse carpal ligament via a small proximal wrist incision.^15^ The use of ultrasound as an adjunct during CTR provides real-time visualization of critical structures, enhancing safety and precision while deploying the device.^9^^,^^10^ Ultrasound allows for accurate identification of the median nerve with surrounding tendons and vessels, aiding in the precise location and release of the transverse carpal ligament. It also provides the ability to assess the severity of median nerve compression and the presence of anatomical variations preoperatively, allowing for better surgical planning.^18^ The use of a small proximal wrist incision in CTR-US minimizes disruption to the palmar tissues, possibly reducing the risk of scar sensitivity, and hand weakness associated with more extensive incisions. The ultrasound can also be a useful point-of-care tool to assess postoperative hematoma, seroma, or abscess and to monitor nerve recovery.

Carpal tunnel release with ultrasound guidance presents a learning curve as surgeons become accustomed to the use of ultrasound guidance for visualization and accurate release. Dekimpe et al found that senior operators learned CTR-US with a hook-knife on a cadaver specimen more quickly than a junior operator.^19^ With increasing availability and familiarity of ultrasound imaging, hand surgeons and plastic surgeons can quickly adapt to incorporating CTR-US into their practice.

### Limitations

There were important differences among the included studies that merit consideration. Surgeries studied by Rojo-Manaute et al^15^ and Capa-Grasa et al^17^ were performed by a single surgeon while de la Fuente et al. had separate surgeons performing CTR-US and OCTR.^15^, ^16^, ^17^ Rojo-Manaute et al and Capa-Grasa et al used blinded outcome assessments, whereas de la Fuente et al did not describe who collected the outcome measures or any blinding procedures. No outcome measures were recorded until 3 months postprocedure in de la Fuente et al’s study, whereas Rojo-Manaute et al and Capa-Grasa et al started data collection by 1 week postprocedure. Given that some of the most important purported benefits of CTR-US are related to the early recovery period, it is possible that the use of a 3-month time point as the first postoperative assessment in de la Fuente et al was inadequate to detect differences between techniques in the early postoperative period. Nonetheless, the authors did report that patients in the CTR-US group had significantly better functional scores at 3 months compared with the OCTR group, although the difference did not reach minimal clinically important difference.

Rigorous systematic review and meta-analysis techniques were applied to reduce bias. Among the included studies, most domains of risk of bias were either low or unclear, with no studies or individual domains at substantial risk of bias (Figs. 5 and 6). This study is limited by the volume of available evidence. Only three RCTs matching the eligibility criteria were available, all of which were conducted in Spain. There are no available studies comparing endoscopic CTR with CTR-US, and thus, we could not assess outcome differences between these two approaches. This limits both the generalizability of the analysis and the precision around the effect size estimates. Given the limited number of included studies, publication bias could not be assessed. Future RCTs with larger sample sizes will help better establish the precise effect size, CIs, and long-term results of CTR-US. In addition, comparisons of CTR-US with other techniques such as endoscopic CTR and thread release would further contextualize CTR-US’s place in the hand surgeon’s armamentarium. Up to now, CTR-US appears to be at least as safe as open surgery, potentially with a lower risk of complications.

Although our systematic review and meta-analysis demonstrate the safety and effectiveness of CTR-US, it is crucial to acknowledge the need for additional long-term outcome data to establish its superiority over other techniques. Long-term studies assessing patient-reported outcomes, recurrence rates, and complication rates beyond the immediate postoperative period are necessary to comprehensively evaluate the durability and effectiveness of CTR-US. Prospective studies with larger sample sizes and longer follow-up periods would help demonstrate the benefits of CTR-US over other techniques.

## Conclusions

Based on the available level 2 evidence, CTR-US is a safe treatment for CTS and may have benefits of better functional outcomes and faster return to normal activities with no difference in complications. Future RCTs with larger sample sizes and longer follow-ups would further elucidate the benefits of CTR-US.

## Conflicts of Interest

Mohit Bhandari received funding by Sonex, Smith and Nephew, BioTraceIT, Bioventus, Ferring, and Acumed. No benefits in any form have been received or will be received by the other authors related directly to this article.
