# Supplementary material for: Carpal Tunnel Release with Ultrasound Guidance Versus Open and Mini-Open Carpal Tunnel Release: A Systematic Review and Meta-Analysis of Randomized Controlled Trials
Source: J Hand Surg Glob Online. 2025 Jan 20;7(2):121–6. doi: 10.1016/j.jhsg.2024.04.001 (PMC11962905; doi:10.1016/j.jhsg.2024.04.001)
Supplement: Appendix 1 [file mmc1.docx]

**Carpal Tunnel Release with Ultrasound Guidance versus Open Carpal Tunnel Release: A Systematic Review and Meta-Analysis of Randomized Controlled Trials**

**APPENDIX 1 –** Literature Search Strategy

1. Carpal Tunnel Syndrome/
2. Carpal Tunnel Release.mp.
3. surgery.ti,ab. or surgical.ti,ab.
4. 1 or 2
5. 3 and 4
6. exp animals/ not humans.sh.
7. 5 not 6
8. (autobiography or bibliography or biography or clinical trial veterinary or comment or congress or dictionary or English abstract or "expression of concern" or festschrift or government publication or lecture or legal case or legislation or letter or news or newspaper article or observational study veterinary or periodical index or personal narrative).pt.
9. 7 not 8
